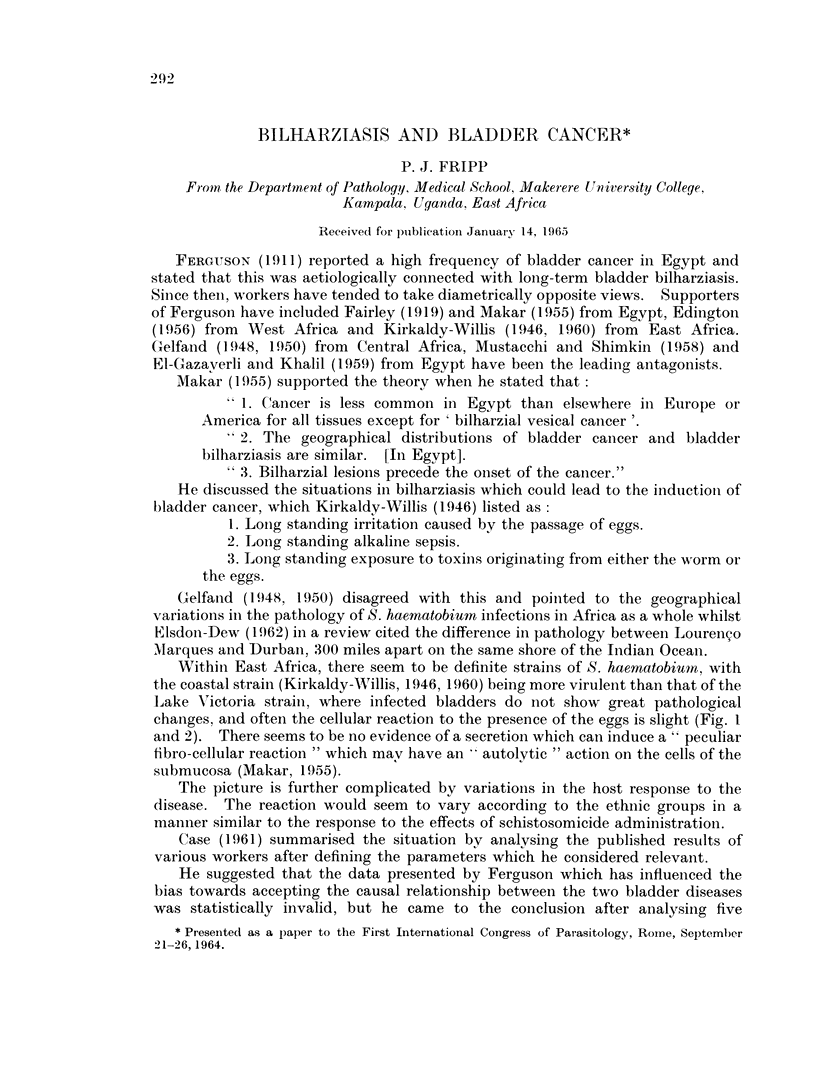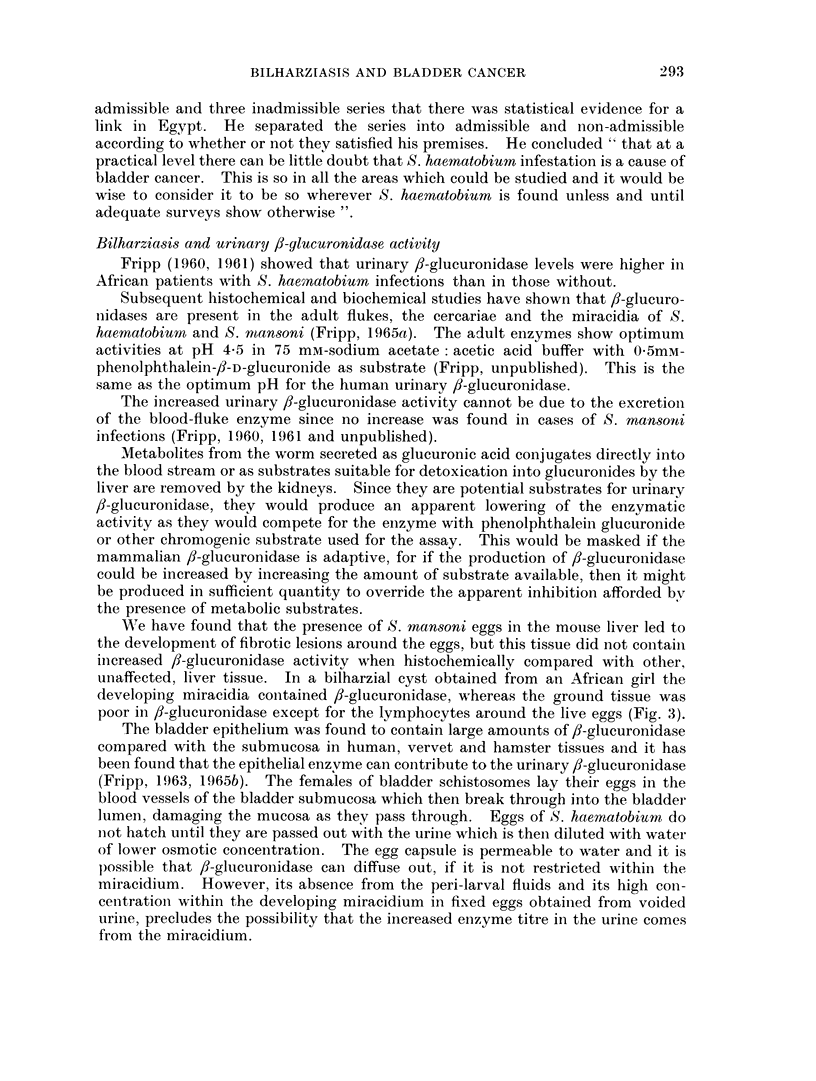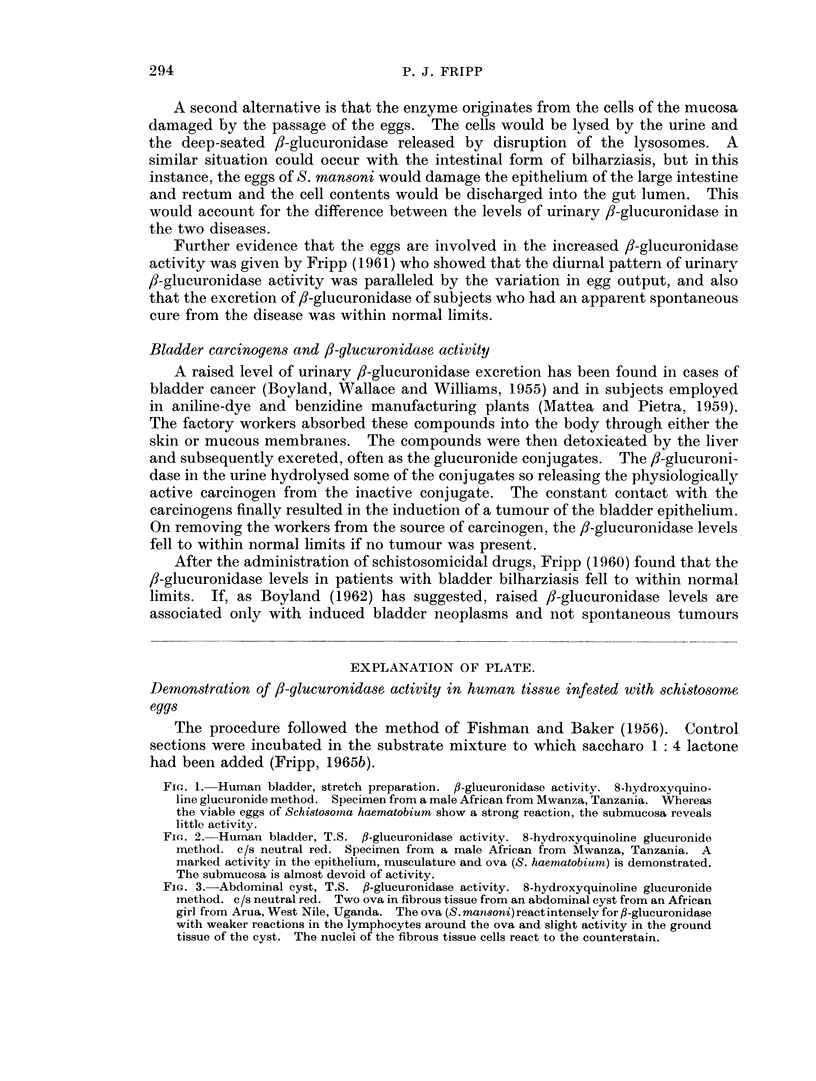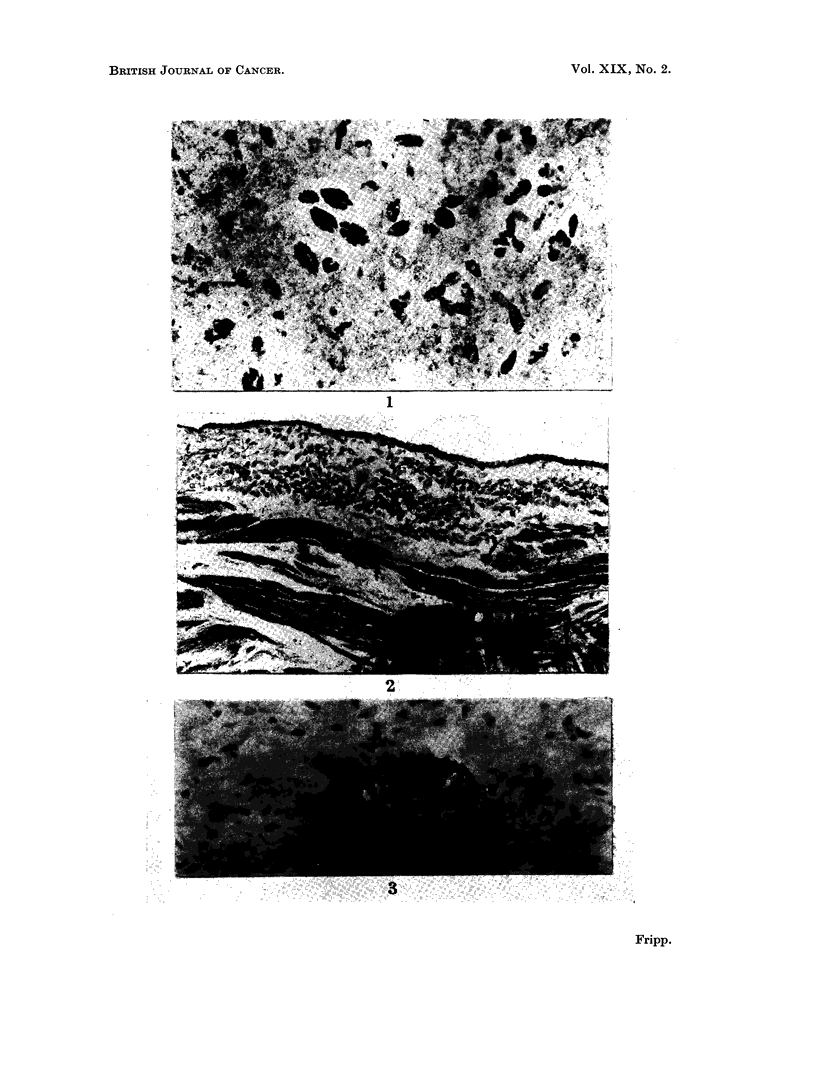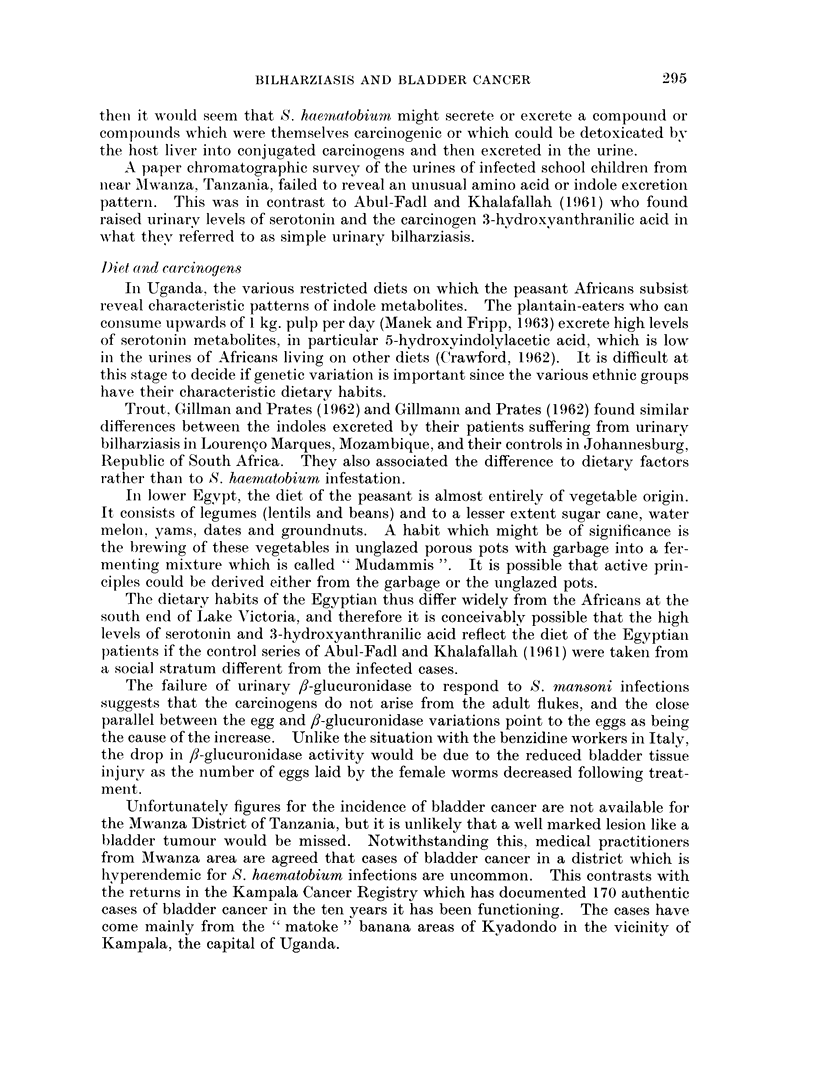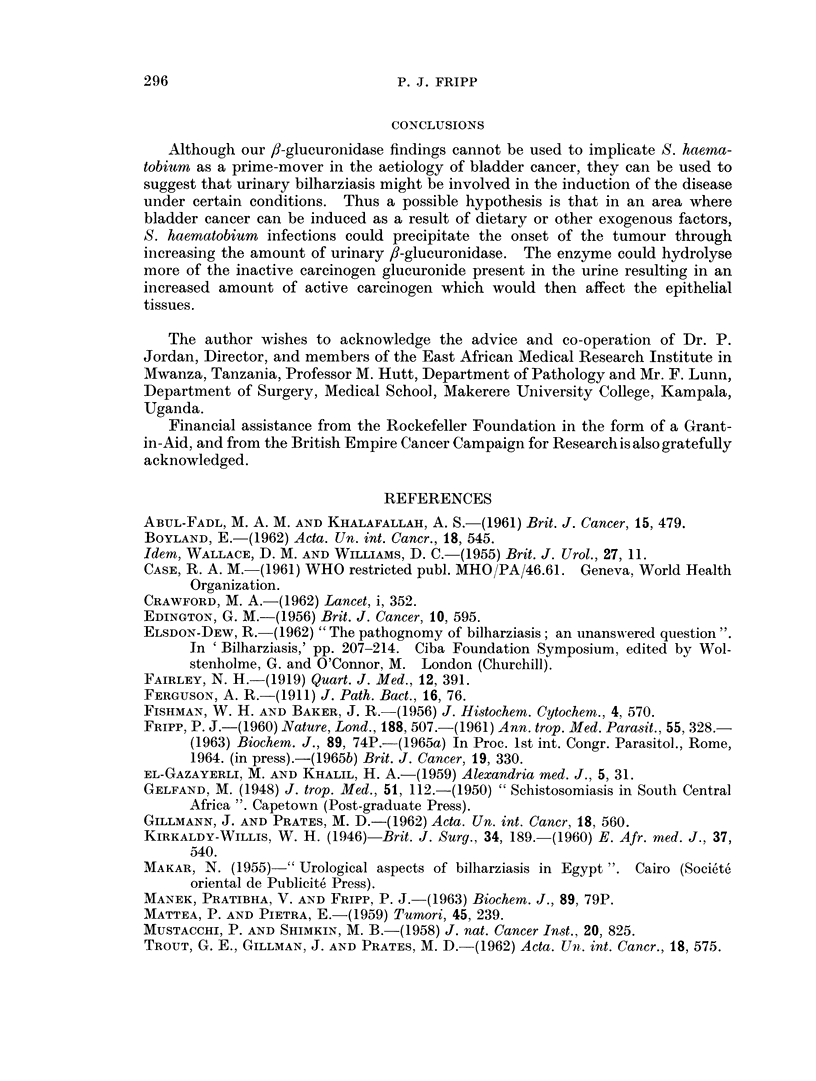# Bilharziasis and Bladder Cancer*

**DOI:** 10.1038/bjc.1965.33

**Published:** 1965-06

**Authors:** P. J. Fripp

## Abstract

**Images:**


### BILHARZIASIS AND BLADDER CANCER*

P. J. FRIPP

*From the Department of Pathology, Medical School, Makerere University College, Kampala, Uganda, East Africa*

Received for publication January 14, 1965

FERGUSON (1911) reported a high frequency of bladder cancer in Egypt and stated that this was aetiologically connected with long-term bladder bilharziasis. Since then, workers have tended to take diametrically opposite views. Supporters of Ferguson have included Fairley (1919) and Makar (1955) from Egypt, Edington (1956) from West Africa and Kirkaldy-Willis (1946, 1960) from East Africa. Gelfand (1948, 1950) from Central Africa, Mustacchi and Shimkin (1958) and El-Gazayerli and Khalil (1959) from Egypt have been the leading antagonists.

Makar (1955) supported the theory when he stated that :

" 1. Cancer is less common in Egypt than elsewhere in Europe or America for all tissues except for ' bilharzial vesical cancer '.

" 2. The geographical distributions of bladder cancer and bladder bilharziasis are similar. [In Egypt].

" 3. Bilharzial lesions precede the onset of the cancer."

He discussed the situations in bilharziasis which could lead to the induction of bladder cancer, which Kirkaldy-Willis (1946) listed as :

1. Long standing irritation caused by the passage of eggs.
2. Long standing alkaline sepsis.
3. Long standing exposure to toxins originating from either the worm or the eggs.

Gelfand (1948, 1950) disagreed with this and pointed to the geographical variations in the pathology of *S. haematobium* infections in Africa as a whole whilst Elsdon-Dew (1962) in a review cited the difference in pathology between Lourenço Marques and Durban, 300 miles apart on the same shore of the Indian Ocean.

Within East Africa, there seem to be definite strains of *S. haematobium*, with the coastal strain (Kirkaldy-Willis, 1946, 1960) being more virulent than that of the Lake Victoria strain, where infected bladders do not show great pathological changes, and often the cellular reaction to the presence of the eggs is slight (Fig. 1 and 2). There seems to be no evidence of a secretion which can induce a " peculiar fibro-cellular reaction " which may have an " autolytic " action on the cells of the submucosa (Makar, 1955).

The picture is further complicated by variations in the host response to the disease. The reaction would seem to vary according to the ethnic groups in a manner similar to the response to the effects of schistosomicide administration.

Case (1961) summarised the situation by analysing the published results of various workers after defining the parameters which he considered relevant.

He suggested that the data presented by Ferguson which has influenced the bias towards accepting the causal relationship between the two bladder diseases was statistically invalid, but he came to the conclusion after analysing five

* Presented as a paper to the First International Congress of Parasitology, Rome, September 21–26, 1964.

admissible and three inadmissible series that there was statistical evidence for a link in Egypt. He separated the series into admissible and non-admissible according to whether or not they satisfied his premises. He concluded " that at a practical level there can be little doubt that *S. haematobium* infestation is a cause of bladder cancer. This is so in all the areas which could be studied and it would be wise to consider it to be so wherever *S. haematobium* is found unless and until adequate surveys show otherwise ".

*Bilharziasis and urinary β-glucuronidase activity*

Fripp (1960, 1961) showed that urinary β-glucuronidase levels were higher in African patients with *S. haematobium* infections than in those without.

Subsequent histochemical and biochemical studies have shown that β-glucuronidases are present in the adult flukes, the cercariae and the miracidia of *S. haematobium* and *S. mansoni* (Fripp, 1965*a*). The adult enzymes show optimum activities at pH 4·5 in 75 mM-sodium acetate : acetic acid buffer with 0·5mM-phenolphthalein-β-D-glucuronide as substrate (Fripp, unpublished). This is the same as the optimum pH for the human urinary β-glucuronidase.

The increased urinary β-glucuronidase activity cannot be due to the excretion of the blood-fluke enzyme since no increase was found in cases of *S. mansoni* infections (Fripp, 1960, 1961 and unpublished).

Metabolites from the worm secreted as glucuronic acid conjugates directly into the blood stream or as substrates suitable for detoxication into glucuronides by the liver are removed by the kidneys. Since they are potential substrates for urinary β-glucuronidase, they would produce an apparent lowering of the enzymatic activity as they would compete for the enzyme with phenolphthalein glucuronide or other chromogenic substrate used for the assay. This would be masked if the mammalian β-glucuronidase is adaptive, for if the production of β-glucuronidase could be increased by increasing the amount of substrate available, then it might be produced in sufficient quantity to override the apparent inhibition afforded by the presence of metabolic substrates.

We have found that the presence of *S. mansoni* eggs in the mouse liver led to the development of fibrotic lesions around the eggs, but this tissue did not contain increased β-glucuronidase activity when histochemically compared with other, unaffected, liver tissue. In a bilharzial cyst obtained from an African girl the developing miracidia contained β-glucuronidase, whereas the ground tissue was poor in β-glucuronidase except for the lymphocytes around the live eggs (Fig. 3).

The bladder epithelium was found to contain large amounts of β-glucuronidase compared with the submucosa in human, vervet and hamster tissues and it has been found that the epithelial enzyme can contribute to the urinary β-glucuronidase (Fripp, 1963, 1965*b*). The females of bladder schistosomes lay their eggs in the blood vessels of the bladder submucosa which then break through into the bladder lumen, damaging the mucosa as they pass through. Eggs of *S. haematobium* do not hatch until they are passed out with the urine which is then diluted with water of lower osmotic concentration. The egg capsule is permeable to water and it is possible that β-glucuronidase can diffuse out, if it is not restricted within the miracidium. However, its absence from the peri-larval fluids and its high concentration within the developing miracidium in fixed eggs obtained from voided urine, precludes the possibility that the increased enzyme titre in the urine comes from the miracidium.

A second alternative is that the enzyme originates from the cells of the mucosa damaged by the passage of the eggs. The cells would be lysed by the urine and the deep-seated $\beta$-glucuronidase released by disruption of the lysosomes. A similar situation could occur with the intestinal form of bilharziasis, but in this instance, the eggs of *S. mansoni* would damage the epithelium of the large intestine and rectum and the cell contents would be discharged into the gut lumen. This would account for the difference between the levels of urinary $\beta$-glucuronidase in the two diseases.

Further evidence that the eggs are involved in the increased $\beta$-glucuronidase activity was given by Fripp (1961) who showed that the diurnal pattern of urinary $\beta$-glucuronidase activity was paralleled by the variation in egg output, and also that the excretion of $\beta$-glucuronidase of subjects who had an apparent spontaneous cure from the disease was within normal limits.

*Bladder carcinogens and $\beta$-glucuronidase activity*

A raised level of urinary $\beta$-glucuronidase excretion has been found in cases of bladder cancer (Boyland, Wallace and Williams, 1955) and in subjects employed in aniline-dye and benzidine manufacturing plants (Mattea and Pietra, 1959). The factory workers absorbed these compounds into the body through either the skin or mucous membranes. The compounds were then detoxicated by the liver and subsequently excreted, often as the glucuronide conjugates. The $\beta$-glucuronidase in the urine hydrolysed some of the conjugates so releasing the physiologically active carcinogen from the inactive conjugate. The constant contact with the carcinogens finally resulted in the induction of a tumour of the bladder epithelium. On removing the workers from the source of carcinogen, the $\beta$-glucuronidase levels fell to within normal limits if no tumour was present.

After the administration of schistosomicidal drugs, Fripp (1960) found that the $\beta$-glucuronidase levels in patients with bladder bilharziasis fell to within normal limits. If, as Boyland (1962) has suggested, raised $\beta$-glucuronidase levels are associated only with induced bladder neoplasms and not spontaneous tumours

---

EXPLANATION OF PLATE.

*Demonstration of $\beta$-glucuronidase activity in human tissue infested with schistosome eggs*

The procedure followed the method of Fishman and Baker (1956). Control sections were incubated in the substrate mixture to which saccharo 1 : 4 lactone had been added (Fripp, 1965*b*).

FIG. 1.—Human bladder, stretch preparation. $\beta$-glucuronidase activity. 8-hydroxyquinoline glucuronide method. Specimen from a male African from Mwanza, Tanzania. Whereas the viable eggs of *Schistosoma haematobium* show a strong reaction, the submucosa reveals little activity.

FIG. 2.—Human bladder, T.S. $\beta$-glucuronidase activity. 8-hydroxyquinoline glucuronide method. c/s neutral red. Specimen from a male African from Mwanza, Tanzania. A marked activity in the epithelium, musculature and ova (*S. haematobium*) is demonstrated. The submucosa is almost devoid of activity.

FIG. 3.—Abdominal cyst, T.S. $\beta$-glucuronidase activity. 8-hydroxyquinoline glucuronide method. c/s neutral red. Two ova in fibrous tissue from an abdominal cyst from an African girl from Arua, West Nile, Uganda. The ova (*S. mansoni*) react intensely for $\beta$-glucuronidase with weaker reactions in the lymphocytes around the ova and slight activity in the ground tissue of the cyst. The nuclei of the fibrous tissue cells react to the counterstain.

1

2

3

Fripp.

then it would seem that *S. haematobium* might secrete or excrete a compound or compounds which were themselves carcinogenic or which could be detoxicated by the host liver into conjugated carcinogens and then excreted in the urine.

A paper chromatographic survey of the urines of infected school children from near Mwanza, Tanzania, failed to reveal an unusual amino acid or indole excretion pattern. This was in contrast to Abul-Fadl and Khalafallah (1961) who found raised urinary levels of serotonin and the carcinogen 3-hydroxyanthranilic acid in what they referred to as simple urinary bilharziasis.

*Diet and carcinogens*

In Uganda, the various restricted diets on which the peasant Africans subsist reveal characteristic patterns of indole metabolites. The plantain-eaters who can consume upwards of 1 kg. pulp per day (Manek and Fripp, 1963) excrete high levels of serotonin metabolites, in particular 5-hydroxyindolylacetic acid, which is low in the urines of Africans living on other diets (Crawford, 1962). It is difficult at this stage to decide if genetic variation is important since the various ethnic groups have their characteristic dietary habits.

Trout, Gillman and Prates (1962) and Gillmann and Prates (1962) found similar differences between the indoles excreted by their patients suffering from urinary bilharziasis in Lourenço Marques, Mozambique, and their controls in Johannesburg, Republic of South Africa. They also associated the difference to dietary factors rather than to *S. haematobium* infestation.

In lower Egypt, the diet of the peasant is almost entirely of vegetable origin. It consists of legumes (lentils and beans) and to a lesser extent sugar cane, water melon, yams, dates and groundnuts. A habit which might be of significance is the brewing of these vegetables in unglazed porous pots with garbage into a fermenting mixture which is called " Mudammis ". It is possible that active principles could be derived either from the garbage or the unglazed pots.

The dietary habits of the Egyptian thus differ widely from the Africans at the south end of Lake Victoria, and therefore it is conceivably possible that the high levels of serotonin and 3-hydroxyanthranilic acid reflect the diet of the Egyptian patients if the control series of Abul-Fadl and Khalafallah (1961) were taken from a social stratum different from the infected cases.

The failure of urinary $\beta$-glucuronidase to respond to *S. mansoni* infections suggests that the carcinogens do not arise from the adult flukes, and the close parallel between the egg and $\beta$-glucuronidase variations point to the eggs as being the cause of the increase. Unlike the situation with the benzidine workers in Italy, the drop in $\beta$-glucuronidase activity would be due to the reduced bladder tissue injury as the number of eggs laid by the female worms decreased following treatment.

Unfortunately figures for the incidence of bladder cancer are not available for the Mwanza District of Tanzania, but it is unlikely that a well marked lesion like a bladder tumour would be missed. Notwithstanding this, medical practitioners from Mwanza area are agreed that cases of bladder cancer in a district which is hyperendemic for *S. haematobium* infections are uncommon. This contrasts with the returns in the Kampala Cancer Registry which has documented 170 authentic cases of bladder cancer in the ten years it has been functioning. The cases have come mainly from the " matoke " banana areas of Kyadondo in the vicinity of Kampala, the capital of Uganda.

### CONCLUSIONS

Although our $\beta$-glucuronidase findings cannot be used to implicate *S. haematobium* as a prime-mover in the aetiology of bladder cancer, they can be used to suggest that urinary bilharziasis might be involved in the induction of the disease under certain conditions. Thus a possible hypothesis is that in an area where bladder cancer can be induced as a result of dietary or other exogenous factors, *S. haematobium* infections could precipitate the onset of the tumour through increasing the amount of urinary $\beta$-glucuronidase. The enzyme could hydrolyse more of the inactive carcinogen glucuronide present in the urine resulting in an increased amount of active carcinogen which would then affect the epithelial tissues.

The author wishes to acknowledge the advice and co-operation of Dr. P. Jordan, Director, and members of the East African Medical Research Institute in Mwanza, Tanzania, Professor M. Hutt, Department of Pathology and Mr. F. Lunn, Department of Surgery, Medical School, Makerere University College, Kampala, Uganda.

Financial assistance from the Rockefeller Foundation in the form of a Grant-in-Aid, and from the British Empire Cancer Campaign for Research is also gratefully acknowledged.

### REFERENCES

ABUL-FADL, M. A. M. AND KHALAFALLAH, A. S.—(1961) *Brit. J. Cancer*, **15**, 479.
BOYLAND, E.—(1962) *Acta. Un. int. Cancr.*, **18**, 545.
*Idem*, WALLACE, D. M. AND WILLIAMS, D. C.—(1955) *Brit. J. Urol.*, **27**, 11.
CASE, R. A. M.—(1961) WHO restricted publ. MHO/PA/46.61. Geneva, World Health Organization.
CRAWFORD, M. A.—(1962) *Lancet*, i, 352.
EDINGTON, G. M.—(1956) *Brit. J. Cancer*, **10**, 595.
ELSDON-DEW, R.—(1962) "The pathognomy of bilharziasis; an unanswered question". In 'Bilharziasis,' pp. 207–214. Ciba Foundation Symposium, edited by Wolstenholme, G. and O'Connor, M. London (Churchill).
FAIRLEY, N. H.—(1919) *Quart. J. Med.*, **12**, 391.
FERGUSON, A. R.—(1911) *J. Path. Bact.*, **16**, 76.
FISHMAN, W. H. AND BAKER, J. R.—(1956) *J. Histochem. Cytochem.*, **4**, 570.
FRIPP, P. J.—(1960) *Nature, Lond.*, **188**, 507.—(1961) *Ann. trop. Med. Parasit.*, **55**, 328.—(1963) *Biochem. J.*, **89**, 74P.—(1965*a*) In Proc. 1st int. Congr. Parasitol., Rome, 1964. (in press).—(1965*b*) *Brit. J. Cancer*, **19**, 330.
EL-GAZAYERLI, M. AND KHALIL, H. A.—(1959) *Alexandria med. J.*, **5**, 31.
GELFAND, M. (1948) *J. trop. Med.*, **51**, 112.—(1950) "Schistosomiasis in South Central Africa". Capetown (Post-graduate Press).
GILLMANN, J. AND PRATES, M. D.—(1962) *Acta. Un. int. Cancr*, **18**, 560.
KIRKALDY-WILLIS, W. H. (1946)—*Brit. J. Surg.*, **34**, 189.—(1960) *E. Afr. med. J.*, **37**, 540.
MAKAR, N. (1955)—"Urological aspects of bilharziasis in Egypt". Cairo (Société oriental de Publicité Press).
MANEK, PRATIBHA, V. AND FRIPP, P. J.—(1963) *Biochem. J.*, **89**, 79P.
MATTEA, P. AND PIETRA, E.—(1959) *Tumori*, **45**, 239.
MUSTACCHI, P. AND SHIMKIN, M. B.—(1958) *J. nat. Cancer Inst.*, **20**, 825.
TROUT, G. E., GILLMAN, J. AND PRATES, M. D.—(1962) *Acta. Un. int. Cancr.*, **18**, 575.